# The Effectiveness and Mechanisms of China’s Grain Support Policies in Relation to Grain Yield—An Evaluation of a Wide Range of Policies

**DOI:** 10.3390/foods14020267

**Published:** 2025-01-15

**Authors:** Tianjian Li, Fan Yang, Haotian Zhang, Qingning Lin

**Affiliations:** 1 College of Economics, Northwest Normal University, No. 967 Anning East Road, Anning District, Lanzhou 730070, China; litianjiannwnu@163.com (T.L.); yf00127@163.com (F.Y.); 13679493427@163.com (H.Z.); 2 Institute of Agricultural Economics and Development, Chinese Academy of Agricultural Sciences, 12 South Avenue, Zhongguancun, Beijing 100081, China

**Keywords:** grain policy, grain production, machine learning

## Abstract

Objective evaluation and in-depth systematic analysis of the effectiveness of implementing a grain support policy series represent an important entry point for improving incentives to grow food, improving grain production support and protection systems, and guaranteeing national food security. Thus, we collected and organized grain support policies during the study period according to the government work reports of 31 provinces in China from 2001 to 2022 and applied a two-way fixed-effects model based on the variables constructed using textual analysis to further explore the effects of a range of grain support policies on grain production gains. The conclusions are as follows: (1) grain support policies significantly contributed to an increase in grain production; (2) grain production gains from grain support policies are more pronounced in less industrialized and disaster-affected areas; (3) a mechanism analysis showed that grain support policies enhanced grain production by expanding the scale of food cultivation, upgrading agricultural mechanization, and strengthening soil erosion control; and (4) further analysis showed that grain support policies increased pesticide use. These conclusions are of great significance for improving grain production support and protection systems, enhancing incentives for farmers to grow food and for local governments to control food, and achieving the goal of food security.

## 1. Introduction

Food security is “the most important thing in the country”. The report of the Twentieth National Congress of the Communist Party of China (CPC) pointed out that food security should be strengthened in all aspects to ensure that the rice bowls of the Chinese are firmly held in their own hands. Since its reform and opening up, China’s grain production has increased from 305 million tons in 1978 to 695 million tons in 2023, feeding approximately 21% of the world’s population from 9% of the world’s arable land [1]. However, the foundation of China’s current food security is not solid, with food security problems caused by agricultural surface pollution and environmental constraints coming to the forefront and resource and market risks accumulating [2]. Globally, the food insecurity situation is undoubtedly exacerbated by large-scale pandemics of new crown epidemics, the Russian–Ukrainian geopolitical conflict, and the frequent occurrence of extreme weather conditions [3]. According to a survey by the Food and Agriculture Organization of the United Nations, approximately 713–757 million people worldwide remained hungry in the year 2023 [4]. China urgently needs to exercise its policy initiative and seek to guarantee national food security at a higher level in view of the international food security situation. Grain policy as exogenous policy support can avoid internal disadvantages and harmonize the interests of all parties, which is an essential means of solving the problem of food security [5]. Therefore, this study explores the effects of China’s grain policy on grain production based on the roles of macro-guidance, micro-incentives, environmental shaping, and resource allocation guidance played by policy in industrial development. Clarifying the specific path of policy intervention in grain production is important for achieving increased grain production and income, solving food security dilemmas, and eradicating world hunger. It is not only a necessary way to enhance China’s food security but also a powerful response to the serious problem of global food security.

Studies have examined the role of input subsidies, factor innovations, price subsidies, and fiscal and financial support on grain production, with the aim of reviewing the existing literature. Agricultural production inputs are substances used or added to the agricultural production process and usually include seeds, fertilizers, pesticides, and other agricultural production materials [6]. Several studies have shown that the use of inputs can significantly enhance grain yield [7,8,9]. Consequently, the use of inputs as a medium to communicate the purpose of policies and promote their effects is common in agriculture, and research assessing the effects of such policies targeting agricultural inputs is widely grounded in the academic community. Zhu et al. [10] explored the effects of China’s input subsidy policy using a sample of Chinese prefecture-level cities from 2007 to 2020, and the empirical study showed that the policy had a significant effect on grain yield increase. Yi et al. [11] concluded that China’s input subsidy policy eased farmers’ liquidity constraints and boosted total grain production by expanding cultivation areas. A study on a large-scale fertilizer subsidy program implemented in Sri Lanka arrived at the same conclusion [12]. Similar to Asian countries, a series of input subsidy schemes (ISPs) adopted by sub-Saharan African countries have likewise proven to be able to significantly boost grain production in African countries. These were designed to provide fertilizers or seeds to farmers at below-market prices and employed precise targeting mechanisms to ensure the proper allocation of resources, and they are still a core component of agricultural development projects in many African countries [13,14]. However, while acknowledging the effect of input subsidy schemes in increasing grain production, some studies also point to the drawbacks of such subsidy schemes, such as crowding out the use of commercial fertilizers, which explains why the actual effect of the policy has been lower than expected [8,10,15]. Ragasa and Mazunda [16] demonstrated that high-quality and well-targeted agricultural extension services could be a useful complement to a highly subsidized system of inputs, with a focus on emphasizing the enormous role played by the new element of science and technology in modern agricultural production [17]. In fact, as early as 1964, Schultz mentioned in his classic book “*Transforming Traditional Agriculture*” that the key to transforming traditional agriculture lies in the introduction of modern factors of agricultural production, which includes investment in human capital such as education and training for farmers. This view is supported by a land distribution policy implemented in Punjab, Pakistan, which showed that land cultivated by farmers with relevant education in the field of agriculture had higher crop yields, with wheat and rice yields increasing by 28.9% and 23.4%, respectively, compared to a control group with no education or training [18]. Innovations in the organizational structure of agriculture, represented by agricultural cooperatives, can also usually lead to increased crop productivity [19,20,21]. These studies confirm that factor innovation plays a significant role in increasing grain production. Grain policies are typically formulated and implemented as price subsidies. On the one hand, price subsidies for food can intervene in the market to stabilize food prices when they fluctuate and protect farmers’ interests. On the other hand, price subsidy policies also have the effect of incentivizing farmers to increase production and thus expand grain production. Relevant studies have shown that price subsidy policies significantly increase grain production [22,23]. Accordingly, this policy has a negative impact, as price support from the government can distort market information and cause a resource mismatch [24], and rising food prices can also put fiscal pressure on the government [25]. Common grain policies include fiscal and financial support. The incentive policy for large grain-producing counties, implemented by the Chinese government in 2005, is an agricultural support policy centered on the form of financial subsidies. This policy aims to provide financial incentives to counties whose grain production reaches a certain standard, which has stimulated the incentives for grain cultivation in areas where the policy has been implemented and increased grain production [26,27,28], thus ensuring national food security. Similarly, the role of financial support policies in the agricultural sector cannot be ignored, and crop insurance has become an important policy tool for the government as a means of reducing agricultural losses and protecting agricultural production [29,30]. Fang et al. [31] confirmed the role of agricultural insurance in increasing total factor productivity in Chinese agriculture, while a study on Polish farms concluded that agricultural insurance could increase land productivity [32]. In the European Union, the Common Agricultural Subsidy Policy (CAP) has boosted agricultural production through improved credit conditions and lower financing costs [33]. The Agricultural Credit Guarantee Fund (ACGF) provided by the Nigerian government, on the other hand, reduces the lending risks faced by commercial banks and promotes agricultural development in the country by alleviating farmers’ credit constraints [34].

However, existing research mostly focuses on the analysis of the effect of a single type of policy and lacks a comprehensive understanding of the policy series as a whole. Thus, ignoring the possible synergistic effect between different types of policies makes it difficult to grasp the policy synergy generated by various grain policies in relation to grain production at the macro-level and to judge the actual effect of the policies. The possible marginal contributions of this study compared to the established research include two main aspects. First, this study updates the research perspective. While previous studies have focused on the impact of a single grain support policy on food production, this paper uses textual analysis to analyze the overall effect of multiple grain support policies from a comprehensive perspective. Second, based on the introduction of the government’s annual growth rate of soil erosion control as a variable in the mechanism analysis, this study updates the path mechanism of grain policy to enhance grain production, determines the relationship between grain policy and grain production, and conducts a more detailed study and exploration of its conduction path, providing theoretical support for the subsequent government’s precise policy implementation.

## 2. Policy Background and Theoretical Analysis

### 2.1. Policy Background

Entering the new century, affected by grain policy and market prices, China’s grain-sowing area was drastically reduced, and grain production continuously declined, with grain production falling short of demand for four consecutive years between 2000 and 2003. Total grain production fell to 860 billion kg in 2003, the lowest point since 1990. In response, the Chinese government introduced a series of policies to benefit farmers and implemented a series of reform measures aimed at stimulating farmers’ incentives to grow food and guarantee national food security. In 2002, the “four subsidies” policy for agriculture, which includes direct grain subsidies, seed subsidies, subsidies for the purchase of agricultural machinery, and comprehensive agricultural subsidies, was implemented, lowering farmers’ costs of growing grain. In 2005, the policy of rewarding counties with large yields was introduced, which effectively increased farmers’ incentives to grow grain by rewarding county-level areas with outstanding grain production performance. In 2006, the Chinese government announced the abolition of the agricultural tax, marking its withdrawal, which had lasted for 2600 years in China. This policy directly reduced the economic burden on farmers, mobilized their motivation to grow food, and promoted grain production. In 2014, China’s rural areas began the reform of the three rights of ownership, and the transfer of land management rights not only promoted the concentration and large-scale operation of land but also facilitated the realization of agricultural mechanization, which ultimately improved the efficiency of agricultural production and grain production. In 2017, the rural revitalization strategy was officially proposed. The rural revitalization strategy is a major decision-making deployment put forward by the Chinese government, aimed at resolving the major historical task of building a moderately prosperous society and a modern socialist country in all respects, and it is the overall focus of the work of the “three rural areas” in the new era. This strategy calls for stabilizing the area under cultivation, strengthening the agricultural infrastructure, and enhancing modern agricultural science and technology, which will enable an increase in China’s grain output through a variety of channels. These policies reflect the will of policymakers to unswervingly support grain production and demonstrate the Chinese government’s determination to guarantee food security. Table 1 provides a brief description of the above policies.

**Figure 1:**
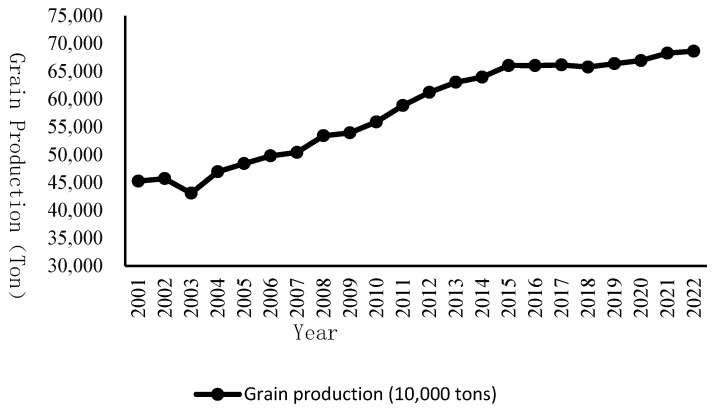
Trends in China’s total grain production, 2001–2022. Note: Data on China’s total grain production from 2001 to 2022 were obtained from official website of National Bureau of Statistics. URL is as follows: https://data.stats.gov.cn/easyquery.htm?cn=C01 (accessed on 13 January 2025).

With the successive implementation of a series of grain support policies, China’s grain production has ushered in a historic phase of continuous growth. Between 2004 and 2015, China’s grain production increased for 12 consecutive years, creating a miracle in the history of grain production worldwide. Since 2015, China’s total annual grain output has consistently remained above 1.3 trillion pounds [35], strongly guaranteeing China’s food security. Figure 1 shows the change in China’s grain production from 2001 to 2022.

### 2.2. Grain Support Policies and Grain Production

Government policies not only have a positioning and guiding role in the development of a certain field but also highlight the great importance the government attaches to that field. The Central Committee of the Communist Party of China (CPC) issued the Central No. 1 Document on Agriculture, Rural Areas, and Farmers for five consecutive years from 1982 to 1986, making specific arrangements for rural reform and agricultural development, and the Central No. 1 Document on the Three Rural Issues (agriculture, rural areas, and farmers) for 21 consecutive years from 2004 to 2024, establishing the importance of these three rural issues in China’s socialist modernization and development. After the government formulated the planning and technical standards, each province, especially the major grain-producing provinces, introduced supporting programs suitable for grain production in their own provinces according to local conditions [36,37,38]. As one of China’s main grain-producing areas, Jiangsu Province has faced a shortage of agricultural water supply in recent years, which has greatly limited local rice production. In response, Jiangsu Province formulated appropriate agricultural water conservation policies under the guidance of the National Agricultural Water Conservation Program. Through the promotion of water-saving irrigation and the implementation of water-use supervision and other measures, the situation of insufficient water for local agriculture has greatly improved, thereby increasing grain production in the province. Based on the excellent local endowment of agricultural resources, the northeast region of China has also formulated policies on japonica rice cultivation and subsidies for the protection of black land in accordance with the actual situation in the region, which has helped give full play to the potential for increasing grain production in the northeast region. Policies often dramatically change social and economic conditions [39], especially in countries such as China, where the government is deeply involved in economic activities [40]. In addition to the direct effect of the policy on grain production, its indirect effect on society should not be overlooked. In areas such as high-quality grain production and soybean revitalization, the government has adopted the “release management service” reform. On the one hand, it has played the role of organizing major projects and government investment, such as the establishment of an investment platform for agricultural development and the establishment of a rural revitalization fund for cooperation between the government and social capital. On the other hand, the government, guided by efficient services and a facilitating environment, has further strengthened the supportive effect of its policies by providing a package of all-round investment services, including planning, project information, financing, land, construction, and operation, for social capital invested in the “three rural areas.” This has allowed the government to unbundle and lighten the burden of private investment and promote the high-quality development of private investment.

Accordingly, this study proposes the first research hypothesis:

**Hypothesis 1 (H1)**. *Grain support policies can promote grain production.*

### 2.3. Mechanism Analysis of Grain Support Policy Affecting Grain Production

#### 2.3.1. Grain Support Policies, Food Cultivation Scale, and Grain Production

It has been shown that the small scale of agricultural operations is one of the important constraints on the increase in agricultural productivity in developing countries [41,42]. In response, the Chinese government implemented a series of measures to expand the scale of grain cultivation and reshape the pattern of agricultural production. The cultivation and development of new types of agricultural management bodies, represented by large specialized households, family farms, and specialized farmers’ cooperatives, are important means of expanding the scale of cultivation. New agricultural management entities have transferred land contract management rights through subcontracting, leasing, swapping, transferring, joint-stock cooperation, and other forms of transfer and have centralized fragmented farmland in rural areas. This has resulted in the realization of “small fields becoming large fields” and “multiple fields becoming one field”, expanding the area of grain cultivation and meeting the needs of new agricultural management entities for continuous farming and large-scale operation. Similarly, the policy on functional grain production zones follows scientific delineation criteria, identifying relatively concentrated and contiguous plots of land with better water and soil resource conditions as functional grain production zones. Under the policy of “basic self-sufficiency in grains and absolute safety in food rations”, the need for large-scale grain cultivation and production in the functional grain production zones has ensured the concentration of plots of land and guaranteed the scale of grain cultivation. These policies have effectively concentrated fragmented land and expanded the scale of food cultivation [43], changing, to some extent, the disadvantages of excessively small and insufficiently concentrated agricultural production in China over thousands of years.

**Figure 2:**
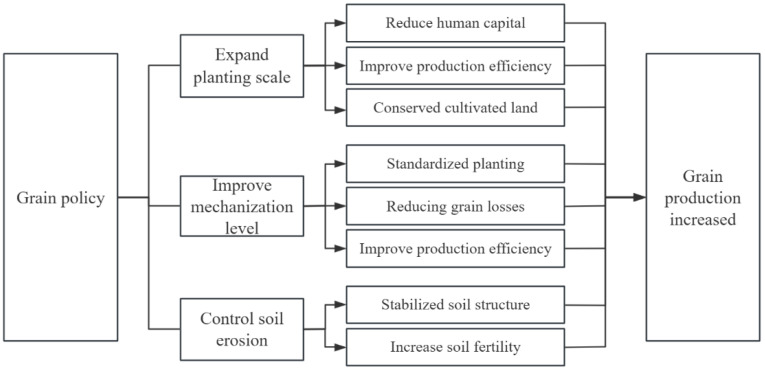
Mechanistic pathways of grain support policies for grain production enhancement.

Expansion of the scale of food cultivation can concentrate fragmented farmland and facilitate mechanized operations, thus reducing human labor and increasing production efficiency [44]. The advantages of large-scale cultivation are also reflected in the efficiency of the use of inputs, such as irrigation water and fertilizers, including irrigation, pesticide sowing, and fertilizer application on large-scale farmland, which reduces wastage and pollution compared to dispersed small plots of land [45]. The consistency and uniformity of standardized operations in the same process also contribute to guaranteeing the quality and quantity of the grains. Finally, the expansion of the scale of food cultivation has created conditions for the realization of crop rotation and fallow. In the past, in the small-scale cultivation environment, farmers could only carry out continuous production for livelihood considerations, and the single fixed planting method not only reduced soil fertility and lowered grain production efficiency but also increased the risk of crops being infected with pests and diseases. Crop rotation and fallow realized on the basis of large-scale planting are not only conducive to the balanced use of soil nutrients and the prevention of diseases, pests, and grass pests but also effectively improve the physicochemical properties of the soil, regulate soil fertility, and ultimately increase production and income. Figure 2 presents the specific mechanism pathway.

Accordingly, this study proposes the second research hypothesis:

**Hypothesis 2 (H2)**. *Grain support policies promote grain output by expanding the scale of grain cultivation.*

#### 2.3.2. Grain Support Policy, Agricultural Mechanization Level, and Grain Production

In 2021, the Ministry of Agriculture and Rural Development issued the “14th Five-Year Plan” for the development of national agricultural mechanization, proposing to make up for the shortcomings of the mechanization of the entire grain production, promote the mechanization of the key aspects of grain production to reduce losses and improve the quality of grain, and build a highly efficient production system for the mechanization of the entire grain industry. It is clear that by 2025, the total power of agricultural machinery nationwide will stabilize at approximately 1.1 billion kilowatts, and the comprehensive mechanization rate of crop plowing, planting, and harvesting will reach 75 percent. It is foreseeable that the mechanization level of China’s agricultural production, especially in the field of grain production, will continue to improve with the strong support of relevant policies. First, the policy has played the role of promotion and demonstration to enhance the level of mechanization [46] by creating a number of overall promotion and demonstration counties in grain production functional areas and national modern agricultural demonstration areas and guiding provinces, cities, counties, and reclamation areas with the conditions to take the lead in realizing the mechanization of the entire grain production industry, and strengthening the role of demonstration and promotion of agricultural machinery. The policy also encourages the organization of participatory, experiential, and interactive agricultural machinery extension “field day” training courses, which allow farmers to visualize the performance and efficiency of agricultural machinery further and greatly enhance the role of extension demonstrations. Second, the policy exerts its effects by providing subsidies to purchase agricultural machinery [47]. This policy aims to provide subsidies in the form of special funds for farmers to purchase agricultural machinery. After selecting and purchasing their own machines, farmers can apply for subsidies from the local agricultural machinery management department and submit the relevant materials. When the application and approval are completed, farmers can obtain subsidies. The policy of subsidizing the purchase of agricultural machinery increased farmers’ willingness and reduced the cost of purchasing agricultural machinery. Finally, the policy provides support to agricultural machinery enterprises through financial support and tax exemptions, encouraging them to research and develop new products, technologies, and processes. The level of production technology and equipment should be improved to provide the market with more new agricultural machinery and equipment adapted to modern agricultural production. Through the above channels, the policy’s effect is greatly reduced, and the level of agricultural mechanization is greatly improved.

Advances in agricultural mechanization are one of the key factors driving a sustained rise in grain production [48]. First, the use of agricultural machinery to achieve precision control in the production process [49], not only to ensure uniform distribution and depth of seeds but also to accurately control the amount of fertilizers and pesticides, improves the efficiency of the use of fertilizers and pesticides, which is conducive to increasing grain yields. Second, the enhancement in the mechanization level can reduce the labor intensity of farmers [50] and improve the efficiency of grain production [51,52]. At the same time, it reduces the dependence of agricultural production on labor and circumvents the risk of grain production reduction caused by an agricultural labor shortage. Finally, the advantages of the high speed and stability of mechanized operations in the harvesting, transportation, and storage of grains are conducive to minimizing losses in natural disaster environments [53], thus enhancing the resilience of the grain production system and guaranteeing food security. Figure 2 presents the specific mechanism pathway.

Accordingly, this study proposes the third research hypothesis:

**Hypothesis 3 (H3)**. *Grain support policies promote grain production by enhancing agricultural mechanization.*

#### 2.3.3. Grain Support Policy, Soil Erosion Control, and Grain Yield

Soil erosion refers to the phenomenon in which, because of the influence of natural or human factors, rainwater cannot be absorbed in situ, flows downhill, and washes away the soil, resulting in the simultaneous loss of water and soil at the same time. This has become an important factor hindering the increase in grain production [54,55]. The Chinese government has made a series of efforts in soil and water erosion control: in 1991, China issued and implemented the Law of the People’s Republic of China on Soil and Water Conservation, which clarified the importance of soil and water control in legal form, and in 2023, the Central Committee of the Communist Party of China (CPC) issued the “Opinions on Strengthening Soil and Water Conservation Work in the New Era”, which further clarified the general requirements and put forward the main objectives of soil and water erosion control work. The above policies not only provide an important basis for China’s soil and water erosion control but also highlight the strong determination of the Chinese government to control soil and water erosion. First, based on sloping arable land with a poor ground level and prominent water, fertilizer, and run-off, which is widely found in the middle and upper reaches of the Yangtze River and the black soil area in the northeast, the policy encourages the management of sloping arable land by means of terracing, furrow management, and changes in cultivation methods. In the Loess Plateau region, the policy calls for the construction of dryland terraces to enhance rainwater harvesting, minimize soil erosion, and develop dryland agriculture. Second, the policy provides support for soil and water erosion control by strengthening the input guarantee. Soil and water erosion control has been developed considerably through the continuous input of funds from central financial authorities and local governments at all levels. Finally, policies should play a leading role in encouraging and supporting the participation of social capital in soil erosion control. When social capital participates in soil and water erosion control, it not only enjoys various support policies from the government but also obtains the right to use natural resources and related rights and interests in accordance with the law after the control work is completed. For example, by carrying out activities such as ecological product development and industrial development, the rights and interests related to soil and water conservation ecological products, such as new arable land and incremental carbon sinks, can be traded in accordance with laws and regulations, and reasonable returns can be obtained. The implementation of these policies is conducive to strengthening China’s soil erosion control in this new era.

As a direct consequence of soil erosion, the structure and stability of the soil are destroyed, and dry and loose soil structures are more susceptible to wind and sand erosion, leading to soil desertification. Soil erosion also results in the loss of nutrients such as nitrogen, phosphorus, and potassium from the soil, with a subsequent decline in soil fertility [56,57], which ultimately affects grain yield and quality. After treatment, the organic matter and water content in the soil increases significantly [58,59], and the soil structure is improved, which ultimately enhances grain yield and quality. Figure 2 presents the specific mechanism pathway.

Accordingly, this study proposes the fourth research hypothesis:

**Hypothesis 4 (H4)**. *Grain support policies promote grain production through erosion control.*

## 3. Research Design

### 3.1. Sample Data

This study selected panel data from 31 provinces in China (excluding Hong Kong, Macau, and Taiwan) from 2001 to 2022 to study the impact of grain support policies on grain production; the data come from China’s National Bureau of Statistics and EPS database. Statistical analysis was performed using Stata 17.0 software. The data were processed in two ways to ensure the accuracy and representativeness of the study: first, the missing values were supplemented using linear interpolation to make the sample size more adequate; second, 1% bilateral shrinkage was implemented for continuous variables to exclude the possible effects of outliers. Finally, 681 sample observations were obtained.

### 3.2. Definition of Variables

#### 3.2.1. Explained Variable: Grain Production

In this study, we referred to Hua et al., (2022) [26] and selected per capita grain production (grain) as an indicator to measure grain production in each province. In the subsequent analysis, we also used three indicators–wheat production (wheat), rice production (rice), and corn production (maize)–to test the robustness of the model. It should be noted that the grain production involved in the variable setting of this paper refers only to China’s domestic production and harvest and does not include food imports.

#### 3.2.2. Explanatory Variables: Grain Support Policies

**Figure 3:**
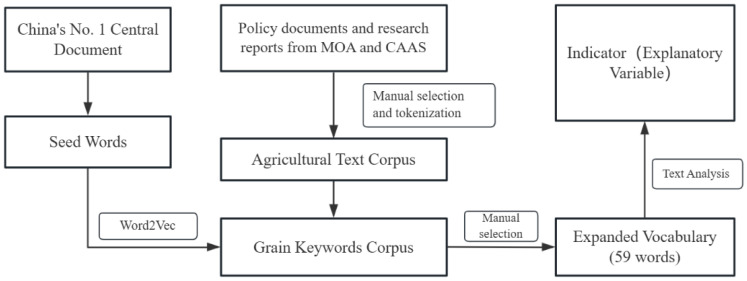
Flowchart for construction of explanatory variable indicators.

Few grain policy studies have directly measured the strength of policy support. This study argued that agriculture, a basic industry in China [60], is a key area of concern for governments everywhere [61]. Consequently, information on grain production characteristics is likely to be reflected in local government policy documents. Among the many policy documents, the government work report will usually be based on central guidance and the actual situation of the province and will clearly put forward the development direction and goals for the coming period. Considering this, this study starts from the perspective of policy text quantification and measures the strength of policy support in each province by extracting and counting keywords related to grain policy in each provincial government’s work report. Figure 3 presents in detail the construction steps for the explanatory variables.

The construction steps of the core explanatory variables in this study are as follows. The first step was to obtain the seed words of grain yield. In this study, we first referred to the No. 1 Central Document, used artificial intelligence to initially extract relevant words about food, and obtained seed words for machine learning through manual screening. The second step was to construct a corpus of agricultural texts. In this study, we manually screened agriculture-related policy documents and research reports from the official websites of the Ministry of Agriculture and Rural Affairs of China and the Chinese Academy of Agricultural Sciences as the original corpus of agricultural texts. They were then processed by word separation and removal of deactivated words to form a corpus of agricultural texts. Third, we constructed a thesaurus of grain policy keywords. Based on the first two steps, this study used the Word2Vec algorithm to expand grain yield words in the agricultural text corpus. The Word2Vec algorithm involves vectorizing the vocabulary of a text context through word embeddings and then calculating the correlation between words based on the cosine similarity of the vectors [62]. In this study, we used the skip-gram model in the Word2Vec model to extract the words that rank in the top 15 in terms of word similarity with those in the database according to the cosine similarity of the computed words and then manually de-emphasized and filtered them to obtain the expanded thesaurus. Finally, the seed words were added to the expanded thesaurus and manually reviewed several times to obtain the final thesaurus of China’s grain support policies, which contains 59 words (as shown in Table 2), of which the top 5 words are “grain” (2594 times), “arable land” (782 times), “food security” (398 times), “basic farmland” (365 times), and “arable land protection” (362 times), which reflects the rationality of keyword selection. The fourth step was conducting a policy text analysis. After the keyword thesaurus was built, we analyzed the text of 681 government work reports of 31 provinces (municipalities and autonomous regions) in China from 2001 to 2022 based on the text quantification method and extracted the number of keyword words for grain production in each government work report. The fifth step was to construct an indicator to measure the strength of policy support. We used the number of grain production keywords plus one to find the logarithm and obtain the explanatory variable grain_policy in this study. The larger the grain policy indicator, the greater the policy support of the region in that year. All of the above steps were carried out in the Jupyter Notebook program using Python code.

#### 3.2.3. Control Variables

Referring to existing studies [61,62,63,64], the control variables selected in this study mainly included the effective irrigation area measured by the logarithm of the effective irrigation area in each province. The total population was expressed as the logarithm of the total population in each province. Gross domestic product (GDP) was expressed as the GDP of each province. Financial support for agriculture (fiscal_agriculture) was expressed as the ratio of expenditure on agriculture, forestry, and water affairs to total fiscal expenditure; disposable income per rural resident (income) was expressed as the logarithm of per capita disposable income in rural areas. The per mu of plastic film used (plastic) was expressed as the ratio of the total amount of plastic film used in agriculture to the total sown area. The degree of disaster (disaster) was expressed as the ratio of disaster-affected area to the total area. The average acre of fertilizer use (fertilizer) was expressed as the ratio of the total amount of fertilizer used in agriculture to the total sown area. The average acre of water use (water) was expressed as the ratio of the total amount of water used in agriculture to the total sown area. The fiscal self-sufficiency rate (fiscal_self) was the ratio of fiscal revenue to fiscal expenditure. For the three-supplement policy (policy), by constructing a dummy variable, we found that if the province has implemented the three-supplement policy in that year, it takes a value of 1 in the current year and the following year; otherwise, it is zero. Electricity use per acre (electricity) was expressed as the ratio of electricity used in agriculture to the total sown area. The level of agricultural technology (agriculture_patent) was expressed by adding one to the total number of agricultural patent applications and taking the logarithm of that value. Table 3 shows the definitional description of all variables.

### 3.3. Descriptive Statistics

The results of the descriptive statistics are shown in Table 4, in which the minimum value of the explanatory variable grain is 0.134, the maximum value is 24.993, the mean is 4.230, and the standard deviation is 3.308, indicating a significant difference between grain production in different provinces. The explanatory variable grain_policy had a minimum value of 0 (After manual review, it was found that the government work reports of Beijing, Tianjin, Shanghai, Guizhou, and Hainan had the phenomenon of not capturing the keywords in individual years, which was due to the fact that the industrial center of gravity of the above regions in that year was not the primary industry. Also, there was no matching vocabulary in the government work reports. So, the explanatory variable was zero.), a maximum value of 3.526, a mean value of 2.066, and a standard deviation of 0.638, indicating large differences in the strength of grain policy support among the sample provinces selected for this study.

### 3.4. Constructing the Model

This study constructed the following model for the benchmark regression to test the impact of grain support policies on grain production:

(1) $${grain}_{it}=\alpha_{0}+\alpha_{1}{grain\_policy}_{it}+\sum_{i=1}^{n}\alpha_{n}{controls}_{it}+\lambda_{i}+\mu_{t}+\varepsilon_{it}$$

where ${grain}_{it}$ is the grain output of province i in year t. ${grain\_policy}_{it}$ is the policy support strength of province *i* in year *t*. ${controls}_{it}$ is a series of control variables, $\lambda_{i}$ is the province fixed effect, and $\mu_{t}$ is the year fixed effect. $\alpha_{1}$ is the regression coefficient of the core explanatory variables.

## 4. Empirical Analysis

### 4.1. Benchmark Regression

The results of the benchmark regression are shown in Table 5, with Columns (1) to (4) denoting the stepwise regression results of including only explanatory variables, adding control variables, controlling for year fixed effects, and controlling for year individual two-way fixed effects, respectively. It can be observed from Table 5 that, after controlling for year and province two-way fixed effects, the coefficient of the explanatory variable grain_policy is 0.232 and is significant at the 1% level; that is, on average, every 1% increase in the intensity of policy support will raise grain output by 0.232%, indicating that grain support policies have a significant positive impact on grain output, and H1 can be verified. It is noted that after controlling for individual province effects, the model parameter estimates drop significantly, implying that there may be an omitted variable problem that ignores individual effects. This also suggests that there may be heterogeneity in the model results in terms of province characteristics.

### 4.2. Endogeneity and Robustness Tests

#### 4.2.1. Instrumental Variables Approach

A reverse causality endogeneity problem may exist between grain support policies and grain production. Specifically, provinces with greater grain policy support may have higher grain production. However, if a province is already a major agricultural province with a focus on grain production, then the local government may provide stronger policy support. Therefore, this study mitigated the endogeneity problem to some extent using the explanatory variable one-period lagged (IV) as an instrumental variable [65], which is estimated using the two-stage least squares (2SLS) method. The regression results are shown in Column (1) of Table 6, where the regression coefficient of instrumental variable IV was significantly positive at the 1% level, indicating a strong correlation between the selected instrumental and explanatory variables. The Kleibergen–Paap rk Wald F statistic is 43.373, which is greater than the critical value of 16.38 at the 10% level, indicating that there are no weak instrumental variables. Concurrently, the Kleibergen–Paap rk LM Wald statistic is significant at the 1% level, rejecting the original hypothesis that instrumental variables are under-identified. The results of the second-stage regression show that the coefficient of the explanatory variable grain_policy is significantly positive at the 5% level, indicating that grain support policies contribute to grain production, which is consistent with the previous analysis.

#### 4.2.2. Replacement of Explanatory Variables

Referring to Hua et al., (2022) [26], this study selected yield wheat, rice yield, and corn yield maize to replace the explanatory variables of grain in the baseline regression. The regression results are shown in Columns (2)–(4) of Table 6, where the coefficients of grain_policy are statistically significant, indicating that the regression results in this study are relatively robust.

#### 4.2.3. Excluding the Impact of the Epidemic

Considering the huge impact of the pandemic on global economic and social activities from 2020 to 2022, including direct and indirect impacts on agricultural production and supply chains, we decided to exclude these three years of data from the robustness test. The regression results are shown in Column (5) of Table 6. The regression results show that the coefficient of grain_policy is 0.143 and is significant at the 5% level, indicating that the regression results in this study are still robust after excluding the effect of epidemic shocks.

#### 4.2.4. Excluding Municipalities

As provincial administrative units, municipalities directly under the central government may differ significantly from other provinces in terms of policy implementation efforts, resource allocation, and administration. These differences may interfere with the relationship between grain support policies and grain production and affect the accuracy of model estimation. Therefore, this study excluded samples from the four municipalities in China for robustness testing. The regression results are presented in Column (6) of Table 6, which shows that the regression coefficient after excluding the municipality samples is 0.203 and is significant at the 5% level, indicating that the benchmark regression results are robust.

### 4.3. Heterogeneity Analysis

#### 4.3.1. Heterogeneity in the Degree of Industrialization

Owing to the significant differences in strategic positioning, natural endowment, history, and culture among the Chinese provinces, the current industrial development status of each province also differs, especially at the level of industrialization. Specifically, provinces with lower levels of industrialization are still in the initial stages of industrial upgrading, and their agricultural production will be more important and will contribute more to grain production. Provinces with higher levels of industrialization have completed their industrial transformation, and agricultural production is relatively less important. In this study, the ratio of the value added of the secondary industry to the total regional output value of each province was used as a criterion to measure the degree of industrialization of the province [26]. If the ratio of a province is higher than the median, the province has a higher degree of industrialization, and vice versa. The results in Columns (1) and (2) of Table 7 show that the enhancement effect of grain support policies on grain production is significant in less industrialized countries. That is, it is significant in provinces in which the importance of the primary sector is higher, with a statistically significant regression coefficient of 0.180.

#### 4.3.2. Heterogeneity of the Degree of Disaster

Agricultural disasters can be categorized as meteorological, biological, or geological disasters. These disasters greatly jeopardize agricultural production by affecting crop growth and destroying farmland and agricultural facilities. They have become one of the most important factors threatening grain production, leading to a reduction in yields. China’s vast territory, complex topography, and susceptibility to monsoons have created conditions conducive to disaster occurrence. Therefore, it is necessary to explore the differential impacts of grain policies in regions with different levels of disaster. In this study, the entire sample was divided into two groups for separate regressions based on differences in the disaster situations experienced by grain production in each province. Specifically, the median success rate (the ratio of the affected area to total area) was used as the threshold. When the success rate is higher than this value, the province has a higher level of food damage in that year, and vice versa. The regression results in Column (3) of Table 7 show that the grain support policy has a stronger effect on grain production enhancement in the more severely affected provinces, with a regression coefficient of 0.203 and significance at the 10% level. The effect of the policy on the less severely affected provinces is 0.097, which is not statistically significant.

### 4.4. Mechanism Analysis

In order to investigate the mechanism of *grain* support policy on *grain* production, this paper constructs the following model:

(2) $$Med=\alpha_{0}+\alpha_{1}grain\_policy+\sum_{i=1}^{n}\alpha_{n}{controls}_{it}+\lambda_{i}+\mu_{t}+\varepsilon_{it}$$

#### 4.4.1. Mechanism Test Results of Expanding the Scale of Grain Cultivation

According to the theoretical analysis in the previous section, the expansion of the grain cultivation scale promotes an increase in grain output by improving production efficiency and maintaining soil fertility. Based on this foundation, this study launched a regression using the grain-sown area of each province as a mechanism variable. Column (2) of Table 8 shows that the coefficient of the explanatory variable is 0.060 and is significant at the 10% level. This result indicates that the grain support policy has been effective in expanding the scale of food cultivation, which, in turn, has a positive effect on grain production. Thus, hypothesis H2 is supported.

#### 4.4.2. The Results of Testing the Mechanism of Increasing the Level of Agricultural Mechanization

This study considered that an increase in the level of agricultural mechanization during grain production is one of the key factors of grain output growth. We used the total power of agricultural machinery in each province as a variable reflecting the level of agricultural mechanization in that province and conducted a regression analysis accordingly. The results are shown in Column (3) of Table 8 The regression coefficient is 0.140, which is significant at the 5% level. This result indicates that the grain support policy significantly enhances the level of agricultural mechanization in the provinces and contributes to an increase in grain production through this mechanism. Thus, H3 is proved.

#### 4.4.3. Strengthening the Mechanism of Soil Erosion Control Test Results

Soil erosion seriously affects agricultural production; however, the harm it causes to agricultural production, especially grain production, is often overlooked. Based on a detailed analysis of soil erosion hazards, this study highlights the importance of effective soil erosion control for increasing grain production. Therefore, we used the erosion control growth rate ((current year’s treated area–previous year’s treated area)/(previous year’s treated area)) as a mechanism variable for the regression to explore the relationship between grain support policies and erosion control. The regression results are presented in Column (4) of Table 8, which suggests that the grain support policy is conducive to improving soil erosion management, which in turn contributes to grain production through this pathway. Thus, H4 is proved.

## 5. Further Analysis

With the development of science and technology in recent times, chemical and biological breakthroughs in the form of pesticides have been widely used in the agricultural production process, greatly improving the growing environment for crops. However, the massive use of pesticides also threatens the ecological environment and human health. The accumulation of non-degradable pesticide pollutants in the air, soil, and water causes soil pollution, water pollution, and biodiversity decline, but pollutants in contact with human beings can also be the causative agent of many diseases, such as cancer, Parkinson’s, and leukemia [66,67,68,69]. Therefore, in recent years, green agriculture, which advocates the reduction in chemical fertilizer and pesticide use and the use of organic fertilizers and biopesticides as an alternative, has attracted much attention [70]. Green agriculture emphasizes the minimization of negative impacts on the environment in the process of agricultural production, focuses on ecological protection and resource conservation, and pursues the “greening” of agricultural production, which is the basic method for implementing a sustainable development strategy and solving the problem of environmental degradation [71].

Pesticide use in China has begun to decline gradually since 2015, when the Chinese government released the Zero Growth Action Program for Pesticide Use by 2020 [72], but globally pesticide use has continued to increase over this period, possibly because increases in pesticide use in other countries and regions have counteracted the reductions brought about by China [73]. Since the 1990s, government policies in Vietnam have promoted the widespread use of pesticides in rice and fruit production [74], and a regional pest control service policy implemented in China has been shown to increase pesticide use [75]. It is evident that the impacts of policies on pesticide usage are still not fully revealed, and this is the contribution that this paper expects to make in further discussions.

In this paper, pesticide use (pesticide) in each province is included in the model for analysis. Pesticide use in each province is used as an explanatory variable to explore the effect of grain policy on pesticide use. The regression results are displayed in Table 9. The results show that the regression coefficient of grain policy on pesticide use is 4.387, and this result is significant at the 1% level, indicating that grain policy brings about an increase in pesticide use. This finding reveals the negative effect of grain policy, which promotes the increase in grain production and at the same time harms the ecological environment and human health by promoting the increase in pesticide use.

## 6. Discussion

This study empirically examined the role of grain support policies in influencing grain production using government work reports of Chinese provinces as the text source and constructed variables using a text analysis method. The benchmark regression results show that grain support policies significantly contributed to an increase in grain production. This is consistent with the findings of Pingali et al., (2015) [76], Wang et al., (2018) [77], and Yuan et al., (2021) [78]. However, the results of some studies, such as those of Pan et al. [79], differ from those of this study, which concluded that the food subsidy policy would cause farmers to develop excessive fertilizer application behaviors, which in turn would cause a decline in grain production. One possible reason for the different conclusions of this study is that compared to the study of the impact of a single policy, we placed the research perspective in a more macro dimension, using the textual source of the government work report to construct a unified variable that encompasses a variety of grain support policies and analyze them holistically, which leads to different conclusions.

The results of the heterogeneity test show that grain support policies exhibit stronger policy effects in severely affected and less industrialized regions. This may be because droughts, floods, and other natural disasters bring about crop failures and extinctions that are beyond the capacity of farmers to resist on their own, greatly affecting grain production. Supportive policies from the government can minimize this negative impact, either through direct subsidies or through the cultivation of arable land, which ultimately improves the situation and increases grain production. A low level of industrialization means that the process of industrial upgrading in the region is slow, and the primary industry, mainly agriculture, is still the mainstay of such regions. Their distinctive features are the vast area of arable land and a large number of agricultural laborers, which indicate that such areas have a long tradition of grain production and strong potential for grain production. As a result, the effects of grain support policies are more fully realized; thus, the increase in grain production in these areas has been significant.

In the mechanism analysis, we explored the possible pathways of the policies’ effect. The results show that grain support policies increase grain production by expanding the scale of food cultivation and upgrading the level of agricultural mechanization, which is consistent with the findings of Wang et al., (2018) [77], Tudi et al., (2021) [66], Peng et al., (2022) [80], and Li (2023) [81]. This study also incorporated the mechanism of governmental erosion control into the analysis and examined its role in the process of grain support policies for promoting grain production. The results show that grain support policies can promote grain production through soil erosion control, a finding that updates the impact pathways of grain policies and provides new ideas for policy formulation and implementation.

In further analysis, this paper explored the possible negative effects of grain policies. The regression results using pesticide use in each province as an explanatory variable show that grain policies have brought about an increase in pesticide use and thus may be harmful to the ecological environment and human health. This finding is conducive to gaining a comprehensive understanding of the effects of grain policies and to formulating improvement plans accordingly.

## 7. Conclusions

This study empirically explored the causal relationship between grain support policies and grain production growth by using government work reports of 31 provinces in China from 2001 to 2022 as the textual source of governmental grain support policies and by adopting a textual analysis method based on machine learning. The results show that grain support policies significantly boosted grain yields, and the mechanism analysis indicates that expanding the scale of grain cultivation, upgrading the level of agricultural mechanization, and strengthening soil erosion control were the pathways through which the policies’ effect was transmitted. Further analysis shows that grain policies can have a negative effect by increasing pesticide use.

First, at a point when Chinese food security is still facing numerous practical challenges, government departments should unswervingly take measures with the aim of “ensuring that the rice bowls of the Chinese people are firmly in their own hands” as a guideline. They should release policy dividends, optimize policy structure, and enhance the effectiveness of policies over a long period to support the increase in grain production and income and to ensure that China’s grain production is enhanced.

Second, government policies should be fully supported to achieve precision and targeting. This study proves that there is a unique relationship between grain support policies and the improvement in grain production. Expanding the scale of grain cultivation, upgrading the level of agricultural mechanization, and strengthening soil erosion control will play a significant role in promoting the effects of the policies, thereby maximizing the sustainable growth of grain production.

Third, grain policies must be tailored to local and layered conditions. It would be helpful if local governments could provide tailor-made grain policies or appropriate policy tilts to areas where agricultural production has been severely affected by disasters and areas with a lower degree of industrialization. By rescuing food disaster losses and tapping into the potential of grain production, policy potential can be enhanced, and policy effects can be expanded. Thus, grain production can be further enhanced, and food security can be guaranteed.

Fourth, while implementing grain policies to increase grain production, the government should take into account the protection of the ecological environment. By promulgating supporting policies to reduce the use of chemical pesticides and promote green agriculture, encouraging the research and development of biological pesticides, and promoting their use to farmers at a lower cost, the negative impact of grain policies on the ecological environment will be avoided to a large extent.

## Figures and Tables

**Table 1:** Grain support policies introduced in China, 2001–present.

Policy	Backdrop	Objective	Measure
“Four subsidies” policy (2002)	Inefficiency in grain production and a lack of enthusiasm among farmers for grain production	Safeguarding the productivity of cultivated land, securing national food security, and enhancing farmers’ income	Subsidies for good crop varieties, agricultural machinery purchase subsidy, direct subsidies to grain producers, and general subsidies for agricultural production supplies
Granary county subsidy program (2005)	The decline in grain production in 2003 and the significant fiscal pressure faced by major grain-producing counties	Mitigating fiscal challenges in counties and townships, stimulating the enthusiasm of local governments to prioritize agriculture and grain production, and promoting the stability and increase in grain output	Financial rewards are provided to both conventional and super grain-producing counties
Abolish the agriculture tax (2006)	Farmers face heavy burdens, insufficient income, and a lack of enthusiasm for grain cultivation	Reduce the cost of agricultural production and management, stimulate the enthusiasm of grain farmers, safeguard national food security, alleviate the burden on farmers, and increase farmers’ income	The complete exemption from agricultural taxes and the abolition of the ‘Agricultural Tax Regulations’
Separating rural land ownership rights, contract rights, and management rights (2014)	Substantial rural labor force migration to urban employment leads to frequent abandonment of contracted farmland	Promote the rational use of land resources, construct a new agricultural management system, enhance land productivity, labor productivity, and resource utilization rates, and increase grain output	Maintaining the collective ownership of rural land, the separation of contractual rights from management rights is implemented, resulting in a structure where ownership rights, contractual rights, and management rights are distinctly separated
The rural vitalization strategy (2017)	Rural–urban development disparity, low income among farmers, and insufficient enthusiasm for grain cultivation	Strengthen the mechanism for food security, enhance the protection and development of arable land, elevate the level of agricultural equipment and informatization, and increase farmers’ income	Fiscal financial support, industrial development support, and guidance for social capital

**Table 2:** Thesaurus of keywords for grain support policies.

Keywords
Food Security, Arable Land, Grain Price Subsidies, Farmland Protection, Mechanized Tillage, Fertilizer Reduction, Inter-Provincial Horizontal Benefit Compensation Mechanism for Grain Production and Marketing Areas, Grain Circulation, Agricultural Disaster Prevention, Mitigation, and Relief, Basic Farmland, Irrigated Area, Agricultural Infrastructure Development, Grain Storage Facilities, Staple Food, Grain Fields, Commercial Grain, Allocation of Grain Production Factors, Grain Warehousing, Production and Demand, Grain Market, Drought and Flood, Scale and Modernization of Grain Production, Improvement of Grain Yield per Hectare, Agricultural Socialized Services, Urban-Rural Economy, Agriculturalizing, Agricultural Machinery and Equipment, Grain Farmers, Modern Agricultural Management System, Contract Farming, Monitoring of Grain Consumption, Smallholder Farmers, Grain Reserves, Smallholder Economy, Family Farming, Medium and Low-Yield Farmland, Grain Prices, Red Line, Grain, Grain Processing, Regulation Capacity for Grain and Important Agricultural Products, Joint Production Contracting, Grain Utilization, Intensive Management, Main Sales, Seed Contracting, Agricultural Technology Support, Mechanical Equipment, Major Grain-Producing Counties, Emphasis on Agriculture, Grainification, High and Stable Yield, Production of Grain and Important Agricultural Products, High Quality and High Price, Food Conservation, Grain Insurance, Commercial Quantity, Eight Consecutive Increases, Revitalization of Seed Industry

**Table 3:** Description of variable definitions.

Variable	Variable Definition
grain	Grain production per capita
grain_policy	Grain yield-related keyword word frequency captured by text analysis and plus one to find its logarithm
irrigation area	Logarithm of effective irrigated area by province
population	Logarithm of total population by province
GDP	Provincial GDP
fiscal_agriculture	Ratio of expenditure on agriculture, forestry, and water affairs to total fiscal expenditure
income	Rural disposable income per capita in logarithmic terms
plastic	Ratio of total agricultural plastic film use to total sown area
disaster	Ratio of affected area to total area
fertilizer	Ratio of total agricultural fertilizer use to total sown area
water	Ratio of total agricultural water use to total sown area
electricity	Ratio of agricultural electricity consumption to total sown area
agriculture_patent	Total number of agricultural patent applications plus one to find its logarithm

**Table 4:** Descriptive statistics.

Variable	Observations	Mean	S.D.	Min	Max
grain	681	4.230	3.308	0.134	24.993
grain_ policy	681	2.066	0.638	0	3.526
irrigation area	681	19.975	15.687	1.285	60.310
population	681	8.096	0.860	5.666	9.385
GDP	681	2.254	1.234	−1.204	4.633
fiscal_agriculture	681	0.094	0.043	0.016	0.185
income	681	8.874	0.762	7.362	10.413
plastic	681	0.017	0.013	0.002	0.061
disaster	681	0.480	0.157	0.028	0.829
fertilizer	681	0.337	0.121	0.128	0.684
water	681	0.031	0.025	0.006	0.131
electricity	681	0.164	0.567	0.004	5.371
agriculture_patent	681	6.130	1.822	0	9.607

**Table 5:** Benchmark regression results.

	(1)	(2)	(3)	(4)
	Grain	Grain	Grain	Grain
grain_policy	1.604 ***	0.877 ***	0.223 ***	0.232 ***
	(0.189)	(0.166)	(0.086)	(0.083)
irrigation_area		0.155 ***	0.222 ***	0.183 ***
		(0.011)	(0.412)	(0.035)
population		−1.684 ***	−2.975 ***	−8.511 ***
		(0.404)	(1.070)	(2.618)
GDP		−0.899 *	−1.634	−2.719 **
		(0.502)	(1.082)	(1.124)
fiscal_agriculture		7.583 ***	6.342	0.850
		(2.204)	(5.156)	(5.313)
income		1.052 *	1.494	1.355
		(0.557)	(1.494)	(1.316)
plastic		−33.741 ***	−42.850 *	−20.481
		(5.596)	(22.318)	(14.950)
disaster		0.606	−0.272	0.053
		(0.566)	(0.267)	(0.279)
fertilizer		−3.120 ***	2.264	2.522
		(0.714)	(2.488)	(2.084)
water		−40.181 ***	−8.470	−14.126
		(4.841)	(22.892)	(5.193)
electricity		0.235 **	0.009	0.119
		(0.093)	(0.064)	(0.109)
agriculture_patent		0.238	−0.273	−0.295
		(0.155)	(0.21)	(0.190)
_cons	0.917 **	6.001	14.031	61.291 **
	(0.409)	(6.668)	(16.507)	(24.915)
N	681	681	681	681
r2	0.096	0.584	0.698	0.735
controls	No	Yes	Yes	Yes
year	No	No	Yes	Yes
province	No	No	No	Yes

Note: *, **, and *** indicate significance at the 10%, 5%, and 1% levels, respectively. Robust standard errors are indicated in parentheses.

**Table 6:** Endogeneity and robustness tests.

	(1)		(2)	(3)	(4)	(5)	(6)
	Grain_Policy	Grain	Wheat	Rice	Maize	Grain	Grain
L.grain_policy	0.267 ***		0.065 **	0.053 **	0.059 *	0.143 **	0.203 **
	(0.041)		(0.030)	(0.024)	(0.035)	(0.064)	(0.095)
grain_policy		0.806 **					
		(0.301)					
irrigation_area	−0.004	0.185 ***	0.083 ***	0.066 ***	0.012 **	0.173 ***	0.168 ***
	(0.006)	(0.035)	(0.011)	(0.010)	(0.005)	(0.036)	(0.030)
population	0.235	−7.850 ***	−1.811 **	−2.141 ***	−1.155 **	−5.389 **	−14.435 ***
	(3.790)	(2.508)	(0.758)	(0.591)	(0.464)	(2.041)	(3.194)
GDP	0.150	−2.544 ***	−1.151 ***	−0.958 **	−0.288	−1.561	−2.298 **
	(0.231)	(0.827)	(0.384)	(0.355)	(0.288)	(0.958)	(0.927)
fiscal_agriculture	−1.487 **	4.510	−0.773	−1.555	0.610	1.438	4.899
	(0.679)	(3.007)	(1.933)	(1.555)	(0.910)	(4.229)	(5.051)
income	0.218	3.773 ***	0.906 *	0.994 *	0.890	2.279	1.505
	(0.248)	(1.110)	(0.518)	(0.549)	(0.597)	(1.452)	(1.224)
plastic	6.927	−23.511	−6.577	−8.748	14.331 *	−19.582	−24.216 *
	(8.996)	(15.468)	(8.021)	(6.204)	(7.166)	(15.792)	(13.233)
disaster	−0.168	0.152	−0.044	−0.068	−0.007	−0.400	−0.142
	(0.107)	(0.238)	(0.096)	(0.087)	(0.070)	(0.245)	(0.290)
fertilizer	−0.318	2.899 *	−0.870	0.075	−1.505	0.080	3.889
	(0.530)	(1.550)	(0.861)	(0.750)	(0.896)	(1.600)	(2.542)
water	−0.946	−3.881	−2.863	1.178	−10.192 **	−4.600	−36.382 *
	(4.113)	(18.481)	(6.983)	(5.610)	(0.043)	(18.400)	(20.909)
electricity	−0.101 **	0.187	−0.005	−0.017	−0.090	−0.028	2.688 **
	(0.049)	(0.117)	(0.238)	(0.023)	(0.058)	(0.093)	(1.088)
agriculture_patent	−0.181 ***	−0.222	−0.100	−0.084	0.020	−0.488 **	−0.262
	(0.040)	(0.172)	(0.074)	(0.053)	(0.041)	(0.202)	(0.200)
_cons			11.254	11.053	7.598	29.680	108.871 ***
			(6.904)	(6.823)	(6.228)	(21.824)	(23.263)
N	649	649	681	681	681	588	594
r2		0.676	0.698	0.781	0.637	0.729	0.782
controls			Yes	Yes	Yes	Yes	Yes
year			Yes	Yes	Yes	Yes	Yes
province			Yes	Yes	Yes	Yes	Yes
Kleibergen–Paap rk Wald F	43.373 [16.38]						
Kleibergen–Paap rk LM Wald	14.323 ***						

Note: *, **, and *** indicate significance at the 10%, 5%, and 1% levels, respectively. Robust standard errors are indicated in parentheses.

**Table 7:** Heterogeneity analysis.

	(1)	(2)	(3)	(4)
grain_policy	0.154	0.180 *	0.203 *	0.097
	(0.096)	(0.097)	(0.118)	(0.078)
irrigation_area	0.178 ***	0.089 ***	0.141 ***	0.215 ***
	(0.038)	(0.032)	(0.025)	(0.042)
population	−6.413 *	−11.060 **	−4.871 **	−8.953 ***
	(3.644)	(4.051)	(2.146)	(2.508)
GDP	−3.532 ***	−4.127 **	−2.620 ***	−3.611 ***
	(1.136)	(1.557)	(0.870)	(0.948)
fiscal_agriculture	13.997 **	−11.153 *	10.015 *	−3.164
	(5.621)	(6.061)	(5.503)	(1.114)
income	4.252 **	2.881	5.111 **	0.901
	(1.825)	(2.797)	(2.166)	(1.126)
plastic	−45.844 **	5.938	−40.663 **	6.063
	(19.425)	(20.476)	(19.836)	(10.276)
disaster	0.267	−0.283	−0.724	−0.051
	(0.342)	(0.242)	(0.664)	(0.638)
fertilizer	3.086	0.699	3.085	1.797
	(2.303)	(3.329)	(2.592)	(1.997)
water	−10.005	−43.422 **	10.367	−21.903
	(21.750)	(20.010)	(15.473)	(16.593)
electricity	1.108	−0.090	0.217	0.151
	(1.539)	(0.095)	(0.192)	(0.116)
agriculture_patent	−0.126	−0.412	−0.327	−0.237
	(0.206)	(0.371)	(0.212)	(0.193)
_cons	22.062	73.470 **	2.534	71.761 ***
	(36.770)	(31.943)	(25.340)	(20.680)
N	341	340	341	340
r^2^	0.716	0.582	0.713	0.828
controls	Yes	Yes	Yes	Yes
year	Yes	Yes	Yes	Yes
province	Yes	Yes	Yes	Yes

Note: *, **, and *** indicate significance at the 10%, 5%, and 1% levels, respectively. Robust standard errors are indicated in parentheses.

**Table 8:** Mechanism analysis.

	(1)	(2)	(3)	(4)
	Grain	Grain_Area	Agri_Mechanize	Soil_Erosion
grain_policy	0.232 ***	0.060 *	0.140 **	0.008 *
	(0.083)	(0.030)	(0.054)	(0.004)
irrigation_area	0.183 ***	0.047 ***	0.078 ***	−0.000
	(0.035)	(0.010)	(0.010)	(0.001)
population	−8.511 ***	−3.612 ***	−4.696 ***	−0.032
	(2.619)	(1.023)	(0.834)	(0.035)
GDP	−2.719 **	−0.701 *	1.301 ***	−0.019
	(1.124)	(0.373)	(0.458)	(0.016)
fiscal_agriculture	0.850	−1.623	−1.065	0.172 *
	(7.001)	(2.459)	(1.936)	(0.099)
income	1.355	0.336	0.338	−0.021
	(1.316)	(0.730)	(0.939)	(0.044)
plastic	−20.481	−12.958 *	−16.283 *	−0.164
	(14.950)	(6.383)	(8.661)	(0.497)
disaster	0.053	0.091	−0.148	0.017
	(0.279)	(0.165)	(0.170)	(0.018)
fertilizer	2.522	0.217	0.263	0.046
	(2.084)	(0.986)	(1.096)	(0.053)
water	−14.126	−6.004	2.231	0.127
	(19.619)	(8.225)	(5.070)	(0.289)
electricity	0.119	−0.007	0.030	0.001
	(0.109)	(0.044)	(0.034)	(0.004)
agriculture_patent	−0.295	−0.067	−0.157	0.011 **
	(0.190)	(0.071)	(0.108)	(0.005)
_cons	61.291 **	27.932 **	34.861 ***	0.397
	(29.915)	(11.937)	(12.105)	(0.503)
N	681	681	681	681
r^2^	0.735	0.711	0.738	0.082
controls	Yes	Yes	Yes	Yes
year	Yes	Yes	Yes	Yes
province	Yes	Yes	Yes	Yes

Note: *, **, *** indicate significance at the 10%, 5%, and 1% levels. Robust standard errors are given in parentheses.

**Table 9:** Impact of grain policies on pesticide use.

	(1)
	Pesticide
grain_policy	4.387 ***
	(1.380)
irrigation_area	1.566 ***
	(0.473)
population	−92.895 **
	(44.877)
GDP	2.434
	(14.318)
fiscal_agriculture	98.136
	(102.225)
income	72.561 **
	(2.08)
plastic	−1425.198 ***
	(34.885)
disaster	−9.374
	(6.291)
fertilizer	342.567 ***
	(85.856)
water	−1233.546 ***
	(401.807)
electricity	3.241
	(1.988)
agriculture_patent	−7.859 **
	(3.119)
_cons	274.941
	(331.254)
N	681
r^2^	0.694
controls	Yes
year	Yes
province	Yes

Note: **, *** indicate significance at the 5%, 1% levels. Robust standard errors are given in parentheses.

## Data Availability

The original contributions presented in the study are included in the article, further inquiries can be directed to the corresponding author.
